# Bridging ancient wisdom and modern technology: an AI and multi-omics framework for three causes tailored treatment in personalized medicine

**DOI:** 10.3389/fmolb.2025.1732340

**Published:** 2025-12-18

**Authors:** Xuewen Diao, Hao Zhang, Shiqi Wang, Qi Zhang, Zulong Wang

**Affiliations:** 1 The First Affiliated Hospital of Henan University of Chinese Medicine Department of Andrology, Zhengzhou, China; 2 The First Clinical Medical School, Henan University of Chinese Medicine, Zhengzhou, China; 3 Faculty of Chinese Medicine, Macau University of Science and Technology, Macao, Macao SAR, China; 4 The First Affiliated Hospital of Henan University of Chinese Medicine Department, Zhengzhou, China

**Keywords:** artificial intelligence, multi-omics, three causes tailored treatment, personalized medicine, integrative medicine

## Abstract

The ‘one-size-fits-all’ therapeutic model is inadequate to address individual patient variability, creating an urgent need for an integrative framework for precision medicine. The ‘Three Causes Tailored Treatment’ (TCTT) principle from traditional Chinese medicine offers a time-tested, holistic blueprint that simultaneously considers the individual, temporal, and environmental dimensions of health. Here, we argue that the synergy of artificial intelligence (AI) and multi-omics technologies is the key to transforming this ancient wisdom into a modern, quantitative clinical paradigm. We demonstrate how multi-omics data provides the foundational layers to quantify the TCTT principle—for instance, using integrated omics (e.g., genomics, proteomics, microbiome) to establish the individual’s molecular baseline (“Who”); chronomics to capture temporal fluxes (“When”); and the exposome to decipher the internalized environmental imprint (“Where”)—while AI-powered multimodal integration models their complex interactions. By synthesizing evidence across the disease continuum, this review provides a translational roadmap for building dynamic clinical decision-support systems, thereby charting a course toward truly personalized, time-sensitive, and context-aware healthcare.

## 1 Introduction

Adverse drug reactions represent a significant global public health challenge. They underscore the limitations of ‘one-size-fits-all’ therapeutic paradigms and highlight the urgent need for precision solutions that address individual variability (Bouvy et al., 2015; Moyer et al., 2019).

Personalized medicine seeks to meet this need by integrating genetic, environmental, and lifestyle factors (Balistreri et al., 2024). This vision is reflected in global initiatives like the “All of Us” research program and the framework of P4 medicine (Hood and Friend, 2011). However, a core challenge remains: the lack of a cohesive framework to operationalize this multidimensional integration in clinical practice.

This integration challenge finds a powerful parallel in ancient wisdom. The ‘Three Causes Tailored Treatment’ (TCTT) principle from traditional Chinese medicine provides a pre-existing, holistic framework. Rooted in the *Huangdi Neijing* and refined through millennia of practice, it is a pragmatic system. It posits that effective intervention must account for the patient’s unique constitution (e.g., Yin-Yang balance), circadian and seasonal rhythms, and geographical-living context. This systems-thinking approach shares conceptual ground with other holistic models, such as Ayurvedic Prakriti and modern chronomedicine (Huang et al., 2022; Potter and Wood, 2020).

We argue that TCTT provides the structural blueprint precision medicine needs. However, its clinical application has been limited by a fundamental issue: its core concepts are qualitative. Diagnoses like “Qi deficiency” rely on subjective observation and lack the objective biomarkers required for modern drug development and reproducible trials. This lack of a quantitative basis has prevented its full scientific validation and clinical translation.

This review articulates a novel path to bridge this gap. We propose that artificial intelligence (AI) and multi-omics technologies are pivotal not only in quantifying the TCTT dimensions but, crucially, in modeling their interactions (Gao et al., 2022; He et al., 2023; Mukherji et al., 2024). Evidence for this integration is emerging, from using AI to model TCM diagnostics to applying multi-omics to decode the molecular basis of traditional phenotypes (Kale et al., 2024; Wang, 2025; Wei et al., 2025).

The feasibility of this approach stems from a direct mapping: individual constitution corresponds to a multi-omic baseline (genome, proteome, metabolome, microbiome); temporal dynamics to chronomics; and environmental context to the exposome and its biological response (e.g., in the epigenome and microbiome).

This review is designed for a broad audience of precision medicine researchers, bioinformaticians, and clinical scientists. It aims to examine how AI and multi-omics can be employed to quantify and interconnect the three causes described in TCTT.

## 2 Integration of the “three causes tailored treatment” principle in the omics era

The TCTT principle offers a structured and holistic framework for personalized medicine. It systematically accounts for an individual’s unique constitution, temporal dynamics, and environmental context. However, a major challenge persists: translating this conceptual framework into a quantitative and clinically actionable paradigm. Here, we define the ‘operationalization’ of TCTT as a process that deconstructs its integrative vision into three discrete, data-driven dimensions. Each dimension addresses a pivotal question in precision medicine. These are: “Who” is being treated (Individual), “When” to intervene (Time), and “Where” the patient is situated in terms of life context (Environment).

Modern omics technologies furnish the data-driven building blocks essential for this operationalization. They deliver high-resolution, molecular-scale measurements, providing the robust evidence needed to objectify and quantify each TCTT dimension. This mapping establishes a clear correspondence:

The ‘Individual’ dimension is operationalized through the comprehensive molecular baseline of a person. This integrates data—from the static genome to the dynamic proteome, metabolome, and microbiome—to answer “Who”. It defines the individual’s unique health status, disease susceptibility, and potential treatment response.

For instance, the multi-omic study by Watanabe et al. demonstrated that such a molecular portrait can move beyond crude clinical metrics like Body Mass Index to precisely define an individual’s unique metabolic health status, identify hidden susceptibilities (e.g., distinguishing metabolically unhealthy individuals within a normal-weight population), and predict their differential responses to lifestyle interventions (Watanabe et al., 2023).

The ‘Time’ dimension is operationalized through temporal multi-omics fluxes (chronomics), capturing rhythmic oscillations (e.g., circadian, seasonal) in molecular profiles. It answers “When” by identifying how biological timing affects health outcomes and therapeutic windows.

As exemplified by a multi-tissue study in mice (Xin et al., 2021), chronomic profiling revealed distinct kinetics in how diurnal transcriptomes and metabolomes in peripheral organs (e.g., liver vs. heart) entrain to inverted feeding schedules. This quantifies how an intervention’s timing is critically linked to its efficacy, providing a molecular basis for chronotherapy.

The ‘Environment’ dimension is operationalized through the internalized environmental imprint, quantified via the exposome and the host’s biological response (e.g., in the epigenome and microbiome). It answers “Where” by decoding how an individual’s specific living context shapes their health trajectory.

A randomized crossover trial on traffic-related air pollution illustrates this approach (Zhang et al., 2022). By integrating personal exposure monitoring with multi-omics profiling, the study quantified how a specific environmental exposure induces systemic inflammation, oxidative stress, and endothelial dysfunction, directly linking a “where” context to molecular and physiological changes.

**FIGURE 1:**
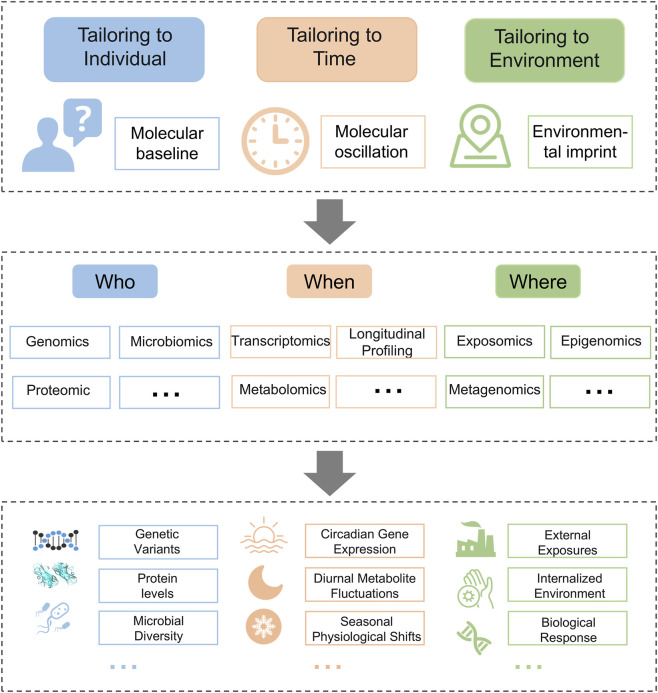
Integration of the ‘three causes tailored treatment’ principle with omics technologies.

The following sections will systematically explore this translation based on the above mapping. We will demonstrate how specific omics technologies generate the data types necessary to parameterize these three critical dimensions, thereby illustrating how the TCTT framework bridges a holistic perspective with the measurable data of modern biology. This Figure 1 visually maps how multi-omics provide the quantitative data streams for the Individual, Time, and Environment dimensions, respectively.

### 2.1 Omics integration for tailoring to individuals: constructing an individual’s multi-omic portrait across the disease care continuum

- The “tailoring to individuals” principle originates from the concept that intervention strategies must account for the patient’s unique constitution. Modern omics technologies operationalize this principle by constructing a quantifiable, multi-omic molecular portrait. This portrait integrates the static genome with dynamic, individually distinctive layers, such as the proteome, metabolome, and microbiome. Together, these layers define an individual’s comprehensive molecular baseline. As illustrated in Figure 1, this integrated portrait enables precision medicine across the entire disease care continuum, from prospective risk stratification and early diagnosis to tailored treatment and dynamic prognosis management.

In the phase of risk assessment and prevention, this portrait begins to take shape, focusing on predictive layers. Omics technologies bridge the gap left by traditional population-average strategies by defining individual risk profiles (Merlo et al., 2017; Pastorino et al., 2021). For example, combining plasma proteomics with polygenic genomic analysis significantly refines colorectal cancer risk stratification (Sun et al., 2024), while specific microbiomic profiles serve as biomarkers to identify high-risk individuals, enabling targeted primary prevention (Frey et al., 2018; Ratiner et al., 2024; Zeybel et al., 2022).

For individuals identified as high-risk, early diagnosis critically relies on detecting active signatures in the molecular portrait. Here, omics technologies move beyond traditional screening by capturing real-time molecular signals with superior sensitivity (Olivier et al., 2019). The application of liquid biopsy combined with omics analysis has proven successful in the early detection of cancers such as pancreatic and ovarian cancer by analyzing circulating genomic and proteomic biomarkers (Zhao Y. et al., 2023; Medina et al., 2024), and shows clear advantages in identifying early metabolomic and proteomic signals of neurological and cardiovascular diseases (Dang et al., 2018; Nurmohamed et al., 2022).

Following diagnosis, personalized treatment strategies leverage the functional and mechanistic dimensions of the portrait. This involves decoding how the individual’s unique biology will interact with therapy. Pharmacogenomics predicts drug sensitivity and resistance by analyzing genomic polymorphisms, such as variations in CYP450 genes that influence warfarin metabolism (Dávila-Fajardo et al., 2019; Manceau et al., 2017). In addition, gut microbiomics plays a crucial role in drug metabolism and overall therapeutic response (Kamble et al., 2024; Zhao Q. et al., 2023). Concurrently, emerging synthetic biology approaches demonstrate the potential for precisely engineering microbial systems. For instance, bacterial strains can be engineered for radiation-induced lysis and the local delivery of therapeutic agents, such as immune checkpoint inhibitors (Green et al., 2019; Kamble et al., 2022). Furthermore, technologies such as single-cell and spatial omics reveal heterogeneity in the disease microenvironment, and metabolomic profiling can identify novel therapeutic targets (Fu et al., 2024; Jiang Y. et al., 2024; Liu B. et al., 2024). Collectively, these insights explain distinct drug responses among patients with similar genetic profiles, thereby guiding the development of personalized combination therapies.

Finally, in prognosis management, the individual’s molecular portrait enables refined risk stratification and outcome prediction. The focus shifts to long-term outcome optimization, which is achieved by leveraging the baseline portrait to forecast future trajectories. Multi-omics analysis can identify poor-prognosis subtypes in acute myeloid leukemia by integrating genomic and transcriptomic features (Liu Y. et al., 2024). Radiomics can predict post-surgical recurrence risk in hepatocellular carcinoma by interpreting tumor phenotype (Kang et al., 2023). Integrated proteomic and immunomic profiling can predict adverse events in immunotherapy (Jing et al., 2020). In this phase, the portrait serves as a definitive map for stratifying patients into distinct management pathways.

- Collectively, by sequentially decoding and integrating individual-specific characteristics across this continuous care cycle, omics technologies complete the data-driven loop of personalized care. The resulting multi-omic portrait is not static but evolves with the individual’s health journey, serving as the foundational compass for all clinical decisions. To ground this discussion in contemporary research, Table 1 summarizes selected studies that exemplify the generation and application of multi-omics data under the ‘Tailoring to Individuals’ principle.

**TABLE 1:** Representative omics studies supporting the ‘tailoring to Individuals’ principle.

Data type	Key findings	Application	References
Proteomics	Developed a model to predict the 10-year risk of 218 diseases	Risk assessment and prevention	Carrasco-Zanini et al. (2024)
cfDNA and proteomics	Achieved high accuracy in early ovarian cancer detection using combined cfDNA and protein biomarkers	Early diagnosis	Medina et al. (2024)
Single-cell sequencing	Revealed drug resistance in lung metastasis; explored combination therapy	Optimizing targeted therapy	Fu et al. (2024)
Multi-omics	Identified a high-risk subgroup (C1) in t (8; 21) AML patients linked to poor prognosis and lower chemotherapy responsiveness	Prognostic stratification	Liu et al. (2024b)
Multi-omics	Predicted immune-related adverse events in PD-1/PD-L1 therapy	Enhancing immunotherapy safety	Jing et al. (2020)

### 2.2 Omics integration for tailoring to time: harnessing temporal dynamics for predictive and adaptive care

The “tailoring to time” principle emphasizes the profound influence of temporal dynamics—from circadian rhythms to seasonal cycles—on health and disease. Modern omics technologies, particularly through the lens of chronomics (temporal multi-omics), operationalize this principle by systematically mapping these biological rhythms. This process converts the abstract concept of time into a quantifiable, molecular data stream (Lincoln et al., 2003). This high-resolution temporal data layer answers the critical question of “When” to intervene, enabling a shift from static to dynamic healthcare across the entire care continuum.

In the realm of prevention, this approach allows for proactive defense aligned with an individual’s biological calendar. Omics analyses can identify individuals with heightened susceptibility to seasonal health threats, such as winter peaks in cardiovascular events, enabling targeted risk mitigation (Dopico et al., 2015; Bhatia et al., 2017). Furthermore, by quantifying the molecular risks of circadian disruption, omics technologies inform the precise timing of interventions (Li Q. et al., 2021). For instance, vaccination schedules or exercise regimens can be personalized to coincide with peaks in immune responsiveness, thereby maximizing their protective efficacy (de Bree et al., 2020; Sato et al., 2022).

For early diagnosis, the temporal dimension refines detection strategies by pinpointing windows of peak molecular signal (Hood and Amir, 2017). The approach moves beyond what to detect to when to detect it. For instance, the metastatic potential of circulating tumor cells peaks during the sleep phase, establishing a temporal window of maximum detectability for liquid biopsy (Diamantopoulou et al., 2022). Similarly, circadian variations in lipid metabolism offer time-specific diagnostic biomarkers for conditions like type 2 diabetes, pointing to optimal morning hours for screening (Sinturel et al., 2023).

The principle finds one of its most direct applications in chronotherapy, where treatment timing is synchronized with biological rhythms to maximize efficacy and minimize toxicity (Plasqui et al., 2003; Tsuruta et al., 2022). Omics data provide the molecular rationale for this synchronization. For example, circadian transcriptomics in cancer is being used to identify temporal windows that improve the efficacy of immunotherapy while reducing side effects (Fang et al., 2015; Sharma et al., 2024). This extends to seasonal rhythms. Omics can illuminate the underlying physiological shifts that occur with the seasons, revealing when therapies need dynamic adjustment. A key example is modifying anticoagulant dosing during seasons when drug clearance is reduced (Lee et al., 2014). Looking forward, the temporal dimension will be equally critical for guiding next-generation therapies. Emerging research, including the use of engineered probiotics for time-specific, epitope-independent radionuclide therapy or for stromal remodeling to enhance immunotherapy, underscores the potential to build temporal control directly into the therapeutic mechanism itself (Siddiqui et al., 2023; Thomas et al., 2022).

Finally, in prognosis management, real-time multi-omics monitoring creates a closed-loop system for adaptive care. The integration of wearable devices with serial molecular profiling generates a continuous stream of time-stamped data, enabling preemptive interventions (Braig, 2022). For example, in organ transplant recipients, single-cell multi-omics can track immune recovery in real-time, guiding precise therapy adjustments (Wang et al., 2024). Similarly, in cancer rehabilitation, longitudinal serum proteome and metabolome profiling allows clinicians to fine-tune nutritional and lifestyle interventions (Braun and Harris, 1983; Li X. et al., 2021). Critically, this dynamic data stream enables chronotherapeutic optimization, such as timing exercise to align with a patient’s daily physiological rhythms to maximize metabolic benefits (Savikj et al., 2022).

This transforms clinical practice from a series of episodic, static decisions into a dynamic, predictive, and adaptive process. This new paradigm respects the intrinsic temporal architecture of health and disease, and will be essential for managing novel and dynamic therapies, such as engineered microbial agents. Table 2 summarizes selected studies that exemplify the generation and application of multi-omics data under the ‘Tailoring to Time’ principle.

**TABLE 2:** Representative omics studies supporting the ‘tailoring to time’ principle.

Data type	Key findings	Application	References
Transcriptomics	Seasonal gene expression changes; winter increases pro-inflammatory markers linked to cardiovascular risks	Seasonal disease risk prediction	Dopico et al. (2015)
Lipidomics	Diurnal lipid metabolism differences in type 2 diabetes vs. non-diabetic individuals, especially in the morning	Time-sensitive disease diagnosis	Sinturel et al. (2023)
Transcriptomics	Circadian patterns in pancreatic cancer cells reveal potential chronotherapeutic targets	Chronotherapy for pancreatic cancer”	Sharma et al. (2024)
Single-cell multi-omics	Real-time immune monitoring post-transplant; guides therapy to reduce rejection	Real-time management of transplant patients	Wang et al. (2024)
Metabolomics and proteomics	Exercise timing influences multi-tissue metabolome and skeletal muscle proteome; afternoon exercise enhances skeletal muscle lipid and mitochondrial content more than morning exercise	Timing exercise for metabolic health	Savikj et al. (2022)

### 2.3 Omics integration for tailoring to environment: decoding the biological imprint of context for precision health

The “tailoring to environment” principle posits that health cannot be understood in isolation from an individual’s geographic, climatic, and lifestyle context. Modern omics technologies put this principle into practice. They do this by systematically reading the biological signals left by environmental exposures, using fields like exposomics and environment-responsive molecular profiling. This profiling turns a person’s surroundings into a quantifiable data layer. This data answers the critical “Where” question of their life context. In doing so, it establishes the final pillar needed for a fully integrated TCTT model.

In risk assessment, this approach moves beyond static genetics to deliver a dynamic, context-aware risk profile. Multi-omics analyses quantify the interplay between genetic susceptibility and environmental exposure. For instance, the integration of geographic exposure mapping, personal sensor data, and multi-omics analysis enables a highly granular understanding of individual risk in inflammatory bowel disease (Agrawal et al., 2022). Similarly, multi-omics approaches can identify and predict cardiovascular risks associated with pollutants, noise, and light exposure, facilitating targeted preventive interventions (Riggs et al., 2018).

For early screening, the focus shifts to the pre-symptomatic detection of environmental and occupational illnesses. This strategy maps the continuum from exposure to preclinical molecular change. In endemic Kashin-Beck disease, exposomics monitoring of regional soil and water, combined with proteomic and metabolomic profiling of individuals, constructs a regional risk atlas for early intervention (Wang et al., 2021). Similarly, for pneumoconiosis, exposomics of workplace dust combined with metabolomic and transcriptomic analysis establishes a molecular signature of early-stage lung injury, enabling action before irreversible damage occurs (Huang et al., 2018; Chen et al., 2022).

Critically, this principle extends to treatment optimization, where omics decode how specific environments alter physiology to guide therapy personalization. For example, omics analyses reveal that high-temperature, humid environments can induce anxiety-like disorders. This effect is mediated through gut microbiota dysbiosis and subsequent metabolic alterations, a discovery that opens avenues for microbiota-targeted interventions (Weng et al., 2024). Similarly, in high-altitude hypoxic environments, omics insights into hypoxia-induced changes in blood-brain barrier protein expression provide a mechanistic basis for devising altitude-adjusted drug administration protocols (Liu G. et al., 2024).

Finally, in prognosis management, the environment itself can be leveraged as a therapeutic modality. Multi-omics data provides the mechanistic basis for prescriptive environmental recommendations (Liu et al., 2023). Exposure to forest environments has been shown to improve cardiopulmonary function, while specific altitude climates can enhance recovery for asthma and coronary heart disease patients (Fieten et al., 2022; Liu et al., 2022; Yan et al., 2024). Furthermore, omics-based analyses elucidate how moderate ultraviolet exposure regulates immune function in autoimmune conditions, enabling the optimization of exposure (Milliken et al., 2012).

In summary, omics technologies quantify how a person’s surroundings affect their biology. This includes harmful exposures and beneficial environments. By adding this dimension, omics complete the integrative vision of TCTT. This empowers a form of precision medicine that is not only personal and timely but also profoundly context-aware, ensuring interventions are optimally tailored to the specific environments in which people live and heal. Table 3 summarizes selected studies that exemplify the generation and application of multi-omics data under the ‘Tailoring to Environment’ principle.

**TABLE 3:** Representative omics studies supporting the ‘tailoring to environment’ principle.

Data type	Key findings	Application	References
Expotomics	Serum exposome reveals toxins linked to chronic diseases; region and age are key factors	Predicting disease risks from environmental exposures	You et al. (2024b)
Microbiome and metabolomics	Gut microbiota changes and altered metabolites linked to Kashin-Beck disease severity	High-risk group identification	Wang et al. (2021)
Multi-omics	Humidity and heat increase psychiatric emergencies; gut microbiota changes contribute to anxiety	Mental health intervention under environmental stress	Weng et al. (2024)
Proteomics	Hypoxia at high altitude affects blood-brain barrier proteins, impacting drug transport	Optimizing drug delivery strategies in hypoxic environments	Liu et al. (2024c)
Metabolomics	Forest NAI exposure enhances HRV and reduces inflammation, benefiting heart health	Cardiovascular prognosis improvement	Liu et al. (2022)

## 3 AI and multimodal models: the computational engine for integrating the three causes tailored treatment

The quantification of the TCTT framework through multi-omics provides the essential data-driven building blocks. However, turning this data into an integrated health model is challenging (Mirza et al., 2019; Saliba et al., 2024). The data types are diverse, ranging from static genomes to dynamic environmental exposures. Furthermore, the interactions between an individual’s biology, temporal rhythms, and environment are complex and nonlinear. Finally, the high-dimensionality of omics data often exceeds the size of typical research cohorts. These characteristics present formidable obstacles for traditional statistical methods. In this context, AI, particularly through multimodal learning, emerges as a powerful computational engine for this integration task, offering a viable path to transform the TCTT framework from a qualitative principle into a quantitative, actionable model (Shao et al., 2023).

### 3.1 Multimodal AI models for three causes tailored treatment

Integrating high-dimensional and heterogeneous “Three-Cause” data demands computational approaches capable of effectively modeling nonlinear relationships and complex interactions, a task that extends beyond the scope of traditional statistical methods. Multimodal artificial intelligence thus serves as the core computational engine for operationalizing the TCTT framework. This section focuses on four key technical architectures that drive multimodal integration: the attention mechanism, graph neural networks, generative models, and encoder-decoder structures (Zhao et al., 2024). The roles of these key AI architectures are summarized in Table 4.

**TABLE 4:** The computational foundation for three causes tailored treatment integration: A comparison of multimodal AI models.

Fusion strategy	Core mechanism	Role in TCTT integration
Encoder-decoder	Projects multimodal inputs into a latent feature space via an encoder and reconstructs or predicts outcomes via a decoder	Facilitates the construction of cross-scale joint representations by embedding heterogeneous data from the “Three-Factor” framework into a unified semantic space
Attention mechanism	Dynamically assigns importance weights to input elements, allowing the model to adaptively attend to the most contextually relevant information	Enables dynamic, context-aware modeling by quantifying the relevance and contribution of each “Three-Factor” component within specific clinical decision-making contexts
Graph neural network	Models data as graphs (nodes represent entities, edges represent relations) and learns node embeddings through neighborhood aggregation (message passing)	Explicitly captures the complex interdependencies and interaction networks among the “Individual-Time-Environment” triad
Generative model	Learns the underlying joint probability distribution of multimodal data to infer deep, intrinsic cross-modal relationships, thereby supporting robust data fusion and synthesis	Enhances robustness and inferential power in data integration, effectively addressing the prevalent issue of incomplete or missing modalities in real-world clinical settings

Cutting-edge research increasingly demonstrates the power of these computational engines to integrate the core dimensions of the TCTT framework.

For instance, an encoder-decoder framework can fuse static individual characteristics with dynamic longitudinal imaging data, thereby jointly modeling the ‘Individual’ and ‘Time’ dimensions within a unified latent space (Gao et al., 2024). This approach enables the dynamic prediction of a patient’s treatment response, moving beyond static assessments. Complementing this, Transformer architectures utilize attention mechanisms to integrate complex multi-omics data. By dynamically weighting the contribution of distinct genomic elements, these models sharpen risk prediction at the ‘Individual’ level, effectively identifying the most salient molecular signals within a high-dimensional dataset (Zhou et al., 2025). Spatial analyses further showcase this integration. Bai et al. employed graph neural networks to explicitly model the interplay between the tumor spatial microenvironment and the host’s molecular characteristics, which form the ‘Individual’ basis of the disease (Bai et al., 2025). Similarly, the generative model soScope enhances data resolution by coherently merging spatial localization (Environment) with molecular profiles (Individual), demonstrating how generative AI can resolve multi-scale biological complexity (Li B. et al., 2024).

In practice, these engines achieve their fullest potential synergistically. A leading example is a high-resolution exposome mapping framework that combines an encoder-decoder structure with an attention mechanism. This integrated strategy successfully fuses multi-source data corresponding directly to the complete ‘individual-time-environment’ triad, providing a powerful blueprint for computational TCTT integration (Luan and Daoyu, 2025).

### 3.2 Towards three causes tailored treatment-informed AI platform: a conceptual framework

**FIGURE 2:**
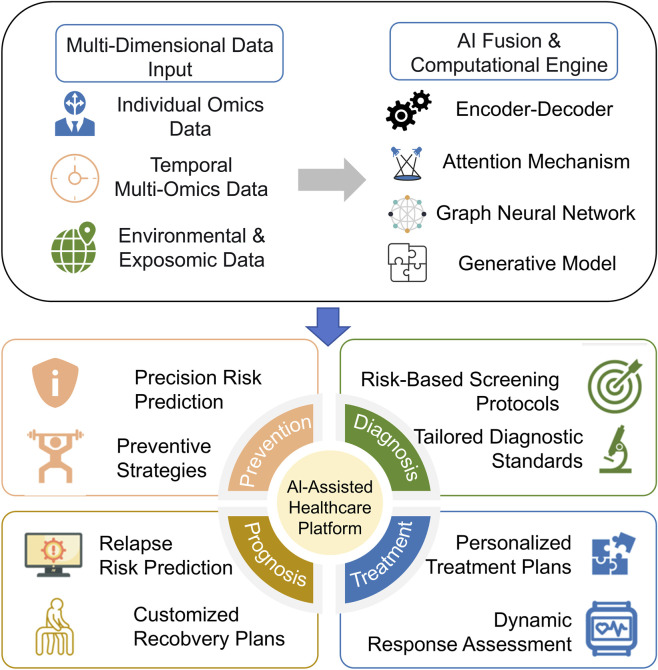
A conceptual framework for three causes tailored treatment-informed precision medicine.

With the widespread adoption of AI in healthcare, AI-assisted platforms are becoming a cornerstone of personalized medicine. Existing platforms, such as IBM Watson Health and Google DeepMind, have demonstrated remarkable potential in areas like medical imaging and diagnostic assistance (Ting et al., 2019; Park et al., 2023). However, these platforms often focus on specific data types or limited clinical applications. To fully realize the vision of the TCTT, we envision a next-generation, comprehensive AI-driven healthcare platform. This conceptual framework, as depicted in Figure 2, is built upon multimodal AI models and omics data, designed to integrate diverse data sources—including genomic, clinical, environmental, and temporal information—to deliver dynamic, context-aware health management.

The proposed framework comprises four key functional modules. Risk Prediction: This module would analyze genetic predispositions (Individual), environmental risk factors (Environment), and seasonal patterns (Time) to offer comprehensive health assessments. For example, integrating omics data with lifestyle information could identify individuals at high risk for diabetes, providing actionable recommendations on diet and exercise that are tailored to their specific circumstances and time of year (Carletti et al., 2025).

Intelligent Diagnosis: Multimodal AI models within this module could enhance diagnostic precision by analyzing diverse datasets. Multimodal models have shown efficacy in evaluating diseases like cancer and Parkinson’s disease, empowering personalized screening strategies for high-risk populations and enabling early interventions (You J. et al., 2024; Liang et al., 2024).

In personalized treatment, multimodal models integrate patient data to predict therapeutic responses and identify optimal treatment regimens. For instance, multimodal analyses have been applied in gastric cancer to predict responses to anti-HER2 therapy and select the most effective treatment plans (Chen et al., 2024). Furthermore, machine learning models might predict circadian time to optimize the timing of drug administration, thereby maximizing efficacy and minimizing toxicity (Hesse et al., 2020).

Prognosis Management: This module envisions closing the loop on continuous, adaptive care by actively managing an individual’s recovery within their environmental context. It would evaluate recurrence risks and tailor follow-up plans by integrating clinical data, omics profiles, and real-time data from wearable devices. For instance, multimodal models can stratify cardiovascular patients into risk groups, allowing for tailored rehabilitation plans that consider environmental factors like air quality and seasonal changes (Castronuovo et al., 2023). This approach is supported by research using multimodal models to stratify patients into risk groups for tailored recurrence management (Jiang Y. Z. et al., 2024).

The synergistic potential of this conceptual framework can be illustrated by envisioning a response to a complex clinical challenge, such as treatment-resistant hypertension. In a future implementation, the platform could first assess a patient’s risk by hypothetically integrating her polygenic score with a gut microbiome signature indicative of impaired bile acid metabolism (Individual) (Xu et al., 2025). If conventional treatment failed, the platform might then analyze longitudinal metabolomic data (Time) to explore whether blood pressure spikes correlate with precursor metabolites, potentially leading to a recommendation for a mechanism-targeted therapy like a bile acid sequestrant. Finally, by incorporating real-time air quality data (Environment), it could generate personalized advice to mitigate environmental risks. This conceptual application demonstrates the TCTT-AI framework’s potential to move beyond siloed data analysis.

It is important to note that the realization of such an integrated platform faces significant challenges, including the technical hurdles of multimodal data fusion, data privacy, standardization, and clinical integration. This conceptual framework thus serves not as a description of an existing tool, but as a roadmap for future research and development, outlining how the TCTT principle can be systematically operationalized through AI to advance precision medicine.

## 4 Challenges and future outlook

The path toward translating the TCTT-informed precision medicine vision into clinical practice is promising yet fraught with multifaceted challenges that span technical, clinical, and socio-ethical domains. While the TCTT framework provides a compelling integrative structure, each of its pillars—individual, time, and environment—faces unique and significant barriers to operationalization that extend beyond the general challenges of precision medicine. Thoughtfully addressing these hurdles is paramount for the framework’s successful integration into mainstream healthcare.

### 4.1 Technical bottlenecks in data integration and modeling

The operationalization of the TCTT framework is primarily hampered by significant technical hurdles. The integration of highly heterogeneous and high-dimensional data across its three core dimensions (individual, time, and environment) presents a formidable obstacle for model development and validation (Jurisica, 2024).

The “Individual” Dimension: Constructing a dynamic multi-omic portrait is not merely a data aggregation exercise. A major limitation is our incomplete understanding of how to distinguish causal drivers of disease from incidental correlations within these vast datasets (Schadt, 2009). For instance, while we can identify microbial signatures associated with disease, determining whether they are a cause or a consequence remains a challenge, complicating targeted interventions.

The “Time” Dimension: Capturing temporal dynamics through chronomics is resource-intensive and analytically complex. Longitudinal sampling at the required frequency (e.g., hourly for circadian rhythms) is impractical for large cohorts and poses a significant patient burden (Price et al., 2017). Furthermore, deconvoluting the effects of aging, disease progression, and reversible rhythmic changes presents a monumental modeling task.

The “Environment” Dimension: Quantifying the exposome is arguably the most nascent pillar. Many environmental exposures are episodic, poorly measured, and interact in complex ways. Current tools often fail to capture the full breadth and personal nature of environmental exposures, from chemical mixtures to psychosocial stressors, limiting the accuracy of this data layer (Wild, 2012).

This “curse of dimensionality” is exacerbated by the relatively small cohort sizes in most current multi-omics studies, increasing the risk of overfitting. Furthermore, the “black-box” nature of many sophisticated AI models raises critical concerns regarding interpretability (Rudin, 2019). For clinicians to trust and act upon AI-generated recommendations, they require understandable rationales. Future efforts must prioritize the development of explainable AI techniques that can elucidate why a specific personalized intervention was recommended, for example, by highlighting that a dosage adjustment was driven 60% by the patient’s pharmacogenomic profile (‘Individual’), 30% by their circadian phase (‘Time’), and 10% by local air quality data (‘Environment').

### 4.2 Data privacy, infrastructure, and standardization

Beyond technical barriers, the framework’s reliance on aggregating sensitive multimodal data inevitably raises profound concerns about patient privacy and data security (Sung, 2023). The implementation of privacy-preserving techniques like federated learning offers a promising path (Li H. et al., 2024). Concurrently, the lack of standardized protocols for data collection across diverse omics platforms and environmental exposures creates significant interoperability issues. This is particularly acute for TCTT, which requires harmonizing data standards across traditionally separate fields—from genomic sequencing to environmental sensor networks. Establishing international consortia and harmonized ethical-regulatory frameworks is imperative to promote data sharing and build the large-scale, diverse datasets needed to train robust and generalizable models.

### 4.3 Clinical translation and validation hurdles

A critical gap lies in proving the clinical utility and cost-effectiveness of this complex approach.

Lack of Trial Evidence: To date, there are no large-scale, prospective clinical trials that simultaneously test interventions tailored to all three TCTT dimensions. The vast combinatorial space of possible “Individual-Time-Environment” interventions makes traditional randomized controlled trials (RCTs) financially and logistically prohibitive.

Novel Trial Designs: Designing trials capable of validating such a dynamic approach is exceptionally difficult (Krassowski et al., 2020). Novel designs, such as adaptive platform trials, N-of-1 series, or “microrandomized” trials, are needed to generate the requisite evidence base.

The Cost-Benefit Question: A stark, unresolved challenge is the overwhelming cost of comprehensive multi-omics profiling and continuous monitoring. The healthcare economics of this approach are unproven, creating a high risk of exacerbating the “biomedical divide,” where such advanced care is only accessible to the affluent (Joyner and Paneth, 2015).

Workflow Integration: Moreover, integrating such a platform into existing clinical workflows poses significant practical challenges. A poorly designed user interface could increase, rather than reduce, the cognitive load on clinicians, hindering adoption (Adams and Petersen, 2016).

### 4.4 Ethical equitability and algorithmic bias

The vision of TCTT-informed precision medicine also introduces pressing ethical considerations. There is a critical risk that AI models could perpetuate or even amplify existing health disparities if they are trained on non-representative datasets primarily from affluent populations, leading to biased and suboptimal outcomes for underrepresented groups (Yang et al., 2024). Proactive measures, such as auditing algorithms for bias and purposefully curating diverse training datasets, are essential to ensure that the benefits of personalized medicine are distributed equitably. Furthermore, the potential for “digital phenotyping” and environmental monitoring to intrude on personal autonomy and lifestyle choices necessitates careful ethical scrutiny and robust informed consent processes. Additionally, the significant costs associated with multi-omics profiling and continuous monitoring raise concerns about economic equity and the potential creation of a “biomedical divide.”

### 4.5 Looking forward: a concerted multi-domain roadmap

Overcoming these hurdles requires a concerted, multi-stakeholder effort. Technological innovation must focus on developing robust, interpretable, and privacy-aware multimodal fusion models. A critical first step is to run pilot studies demonstrating proof-of-concept for individual TCTT dimensions, such as a trial showing that time-of-day-specific dosing (Time) improves outcomes in a genetically stratified (Individual) subgroup. Clinicians, researchers, TCM experts, and regulators must collaborate to establish new standards for data, evidence generation, and the bio-digital translation of traditional concepts. Ethicists and policymakers must work alongside technologists to create governance frameworks that safeguard against bias, protect individual rights, and promote equitable access. By viewing these challenges not as insurmountable barriers but as defined research and implementation priorities, the field can systematically build the technical, ethical, and collaborative foundations needed to realize the full potential of TCTT-driven precision medicine, ultimately delivering on the promise of healthcare that is truly personal, predictive, and participatory.

## References

[B1] Adams S. A. Petersen C. (2016). Precision medicine: opportunities, possibilities, and challenges for patients and providers. J. Am. Med. Inf. Assoc. JAMIA 23, 787–790. 10.1093/jamia/ocv215 26977101 PMC9396673

[B2] Agrawal M. Allin K. H. Petralia F. Colombel J. F. Jess T. (2022). Multiomics to elucidate inflammatory bowel disease risk factors and pathways. Nat. Reviews. Gastroenterology and Hepatology 19, 399–409. 10.1038/s41575-022-00593-y 35301463 PMC9214275

[B3] Bai Z. Osman M. Brendel M. Tangen C. M. Flaig T. W. Thompson I. M. (2025). Predicting response to neoadjuvant chemotherapy in muscle-invasive bladder cancer via interpretable multimodal deep learning. NPJ Digital Medicine 8, 174. 10.1038/s41746-025-01560-y 40121304 PMC11929913

[B4] Balistreri C. R. Vinciguerra C. Magro D. Di Stefano V. Monastero R. (2024). Towards personalized management of myasthenia gravis phenotypes: from the role of multi-omics to the emerging biomarkers and therapeutic targets. Autoimmun. Reviews 23, 103669. 10.1016/j.autrev.2024.103669 39426579

[B5] Bhatia S. Bhatia S. Mears J. Dibu G. Deshmukh A. (2017). Seasonal periodicity of ischemic heart disease and heart failure. Heart Fail Clin. 13, 681–689. 10.1016/j.hfc.2017.05.004 28865777

[B6] Bouvy J. C. De Bruin M. L. Koopmanschap M. A. (2015). Epidemiology of adverse drug reactions in Europe: a review of recent observational studies. Drug Safety 38, 437–453. 10.1007/s40264-015-0281-0 25822400 PMC4412588

[B7] Braig Z. V. (2022). Personalized medicine: from diagnostic to adaptive. Biomed. J. 45, 132–142. 10.1016/j.bj.2019.05.004 35590431 PMC9133264

[B8] Braun D. P. Harris J. E. (1983). Serial immune function testing to predict clinical disease relapse in patients with solid tumors. Cancer Immunol. Immunother. 15, 165–171. 10.1007/BF00199159 6352007 PMC11039147

[B9] Carletti M. Pandit J. Gadaleta M. Chiang D. Delgado F. Quartuccio K. (2025). Multimodal AI correlates of glucose spikes in people with normal glucose regulation, pre-diabetes and type 2 diabetes. Nat. Medicine 31, 3121–3127. 10.1038/s41591-025-03849-7 40745404 PMC12443610

[B10] Carrasco-Zanini J. Pietzner M. Davitte J. Surendran P. Croteau-Chonka D. C. Robins C. (2024). Proteomic signatures improve risk prediction for common and rare diseases. Nat. Medicine 30, 2489–2498. 10.1038/s41591-024-03142-z 39039249 PMC11405273

[B11] Castronuovo G. Favia G. Telesca V. Vammacigno A. (2023). Analyzing the interactions between environmental parameters and cardiovascular diseases using random forest and SHAP algorithms. Rev. Cardiovascular Medicine 24, 330. 10.31083/j.rcm2411330 39076440 PMC11262455

[B12] Chen Z. Shi J. Zhang Y. Zhang J. Li S. Guan L. (2022). Screening of serum biomarkers of coal workers' pneumoconiosis by metabolomics combined with machine learning strategy. Int. J. Environ. Res. Public Health 19, 7051. 10.3390/ijerph19127051 35742299 PMC9222502

[B13] Chen Z. Chen Y. Sun Y. Tang L. Zhang L. Hu Y. (2024). Predicting gastric cancer response to anti-HER2 therapy or anti-HER2 combined immunotherapy based on multi-modal data. Signal Transduction Targeted Therapy 9, 222. 10.1038/s41392-024-01932-y 39183247 PMC11345439

[B14] Dang V. T. Huang A. Werstuck G. H. (2018). Untargeted metabolomics in the discovery of novel biomarkers and therapeutic targets for atherosclerotic cardiovascular diseases. Cardiovasc Hematol. Disord. Drug Targets 18, 166–175. 10.2174/1871529X18666180420170108 29683098

[B15] Dávila-Fajardo C. L. Díaz-Villamarín X. Antúnez-Rodríguez A. Fernández-Gómez A. E. García-Navas P. Martínez-González L. J. (2019). Pharmacogenetics in the treatment of cardiovascular diseases and its current progress regarding implementation in the clinical routine. Genes (Basel) 10, 261. 10.3390/genes10040261 30939847 PMC6523655

[B16] de Bree L. C. J. Mourits V. P. Koeken V. A. Moorlag S. J. Janssen R. Folkman L. (2020). Circadian rhythm influences induction of trained immunity by BCG vaccination. J. Clin. Invest 130, 5603–5617. 10.1172/JCI133934 32692732 PMC7641012

[B17] Diamantopoulou Z. Castro-Giner F. Schwab F. D. Foerster C. Saini M. Budinjas S. (2022). The metastatic spread of breast cancer accelerates during sleep. Nature 607, 156–162. 10.1038/s41586-022-04875-y 35732738

[B18] Dopico X. C. Evangelou M. Ferreira R. C. Guo H. Pekalski M. L. Smyth D. J. (2015). Widespread seasonal gene expression reveals annual differences in human immunity and physiology. Nat. Communications 6, 7000. 10.1038/ncomms8000 25965853 PMC4432600

[B19] Fang L. Yang Z. Zhou J. Tung J. Y. Hsiao C. D. Wang L. (2015). Circadian clock gene CRY2 degradation is involved in chemoresistance of colorectal cancer. Mol. Cancer Ther. 14, 1476–1487. 10.1158/1535-7163.MCT-15-0030 25855785 PMC4458447

[B20] Fieten K. B. Drijver-Messelink M. T. Cogo A. Charpin D. Sokolowska M. Agache I. (2022). Alpine altitude climate treatment for severe and uncontrolled asthma: an EAACI position paper. Allergy 77, 1991–2024. 10.1111/all.15242 35113452 PMC9305916

[B21] Frey A. M. Ansbro K. Kamble N. S. Pham T. K. Stafford G. P. (2018). Characterisation and pure culture of putative health-associated oral bacterium BU063 (tannerella sp. HOT-286) reveals presence of a potentially novel glycosylated S-layer. FEMS Microbiology Letters 365. 10.1093/femsle/fny180 30052903

[B22] Fu M. Zhao J. Zhang L. Sheng Z. Li X. Qiu F. (2024). Overcoming tyrosine kinase inhibitor resistance in lung cancer brain metastasis with CTLA4 blockade. Cancer Cell 42, 1882–1897.e7. 10.1016/j.ccell.2024.09.012 39423817

[B23] Gao P. Shen X. Zhang X. Jiang C. Zhang S. Zhou X. (2022). Precision environmental health monitoring by longitudinal exposome and multi-omics profiling. Genome Research 32, 1199–1214. 10.1101/gr.276521.121 35667843 PMC9248886

[B24] Gao Y. Ventura-Diaz S. Wang X. He M. Xu Z. Weir A. (2024). An explainable longitudinal multi-modal fusion model for predicting neoadjuvant therapy response in women with breast cancer. Nat. Communications 15, 9613. 10.1038/s41467-024-53450-8 39511143 PMC11544255

[B25] Green C. A. Kamble N. S. Court E. K. Bryant O. J. Hicks M. G. Lennon C. (2019). Engineering the flagellar type III secretion system: improving capacity for secretion of recombinant protein. Microb. Cell Factories 18, 10. 10.1186/s12934-019-1058-4 30657054 PMC6337784

[B26] He X. Liu X. Zuo F. Shi H. Jing J. (2023). Artificial intelligence-based multi-omics analysis fuels cancer precision medicine. Semin. Cancer Biol. 88, 187–200. 10.1016/j.semcancer.2022.12.009 36596352

[B27] Hesse J. Malhan D. Yalҫin M. Aboumanify O. Basti A. Relógio A. (2020). An optimal time for treatment-predicting circadian time by machine learning and mathematical modelling. Cancers 12, 3103. 10.3390/cancers12113103 33114254 PMC7690897

[B28] Hood S. Amir S. (2017). Neurodegeneration and the circadian clock. Front. Aging Neurosci. 9, 170. 10.3389/fnagi.2017.00170 28611660 PMC5447688

[B29] Hood L. Friend S. H. (2011). Predictive, personalized, preventive, participatory (P4) cancer medicine. Nat. Reviews. Clin. Oncology 8, 184–187. 10.1038/nrclinonc.2010.227 21364692

[B30] Huang R. Yu T. Li Y. Hu J. (2018). Upregulated has-miR-4516 as a potential biomarker for early diagnosis of dust-induced pulmonary fibrosis in patients with pneumoconiosis. Toxicol. Res. (Camb) 7, 415–422. 10.1039/c8tx00031j 30090591 PMC6060724

[B31] Huang Z. Chavda V. P. Bezbaruah R. Uversky V. N. P S. Patel A. B. (2022). An ayurgenomics approach: prakriti-based drug discovery and development for personalized care. Front. Pharmacology 13, 866827. 10.3389/fphar.2022.866827 35431922 PMC9011054

[B32] Jiang Y. Lam S. M. Zhang S. Miao H. Zhou Y. Zhang Q. (2024a). CSF multi-omics of intracerebral hemorrhage from onset to reperfusion underscores lipid metabolism in functional outcome. Sci. Bull. (Beijing) 70, 162–166. 10.1016/j.scib.2024.06.005 38971657

[B33] Jiang Y. Z. Ma D. Jin X. Xiao Y. Yu Y. Shi J. (2024b). Integrated multiomic profiling of breast cancer in the Chinese population reveals patient stratification and therapeutic vulnerabilities. Nat. Cancer 5, 673–690. 10.1038/s43018-024-00725-0 38347143

[B34] Jing Y. Liu J. Ye Y. Pan L. Deng H. Wang Y. (2020). Multi-omics prediction of immune-related adverse events during checkpoint immunotherapy. Nat. Communications 11, 4946. 10.1038/s41467-020-18742-9 33009409 PMC7532211

[B35] Joyner M. J. Paneth N. (2015). Seven questions for personalized medicine. Jama 314, 999–1000. 10.1001/jama.2015.7725 26098474

[B36] Jurisica I. (2024). Explainable biology for improved therapies in precision medicine: AI is not enough. Best practice and research. Clin. Rheumatology 38, 102006. 10.1016/j.berh.2024.102006 39332994

[B37] Kale M. Wankhede N. Pawar R. Ballal S. Kumawat R. Goswami M. (2024). AI-driven innovations in alzheimer's disease: integrating early diagnosis, personalized treatment, and prognostic modelling. Ageing Research Reviews 101, 102497. 10.1016/j.arr.2024.102497 39293530

[B38] Kamble N. Kharofa J. R. Vatner R. E. Sertorio M. G. B. Kotagiri N. (2022). Radiolytic *Escherichia coli* nissle: a novel radiosensitizer delivery platform using a live bacterial therapeutic. Int. J. Radiat. Oncol. Biol. Phys. 114, S12. 10.1016/j.ijrobp.2022.07.352

[B39] Kamble N. S. Bera S. Bhedase S. A. Gaur V. Chowdhury D. (2024). Review on applied applications of microbiome on human lives, 3, 141–159.

[B40] Kang W. Cao X. Luo J. (2023). Effect of multiple peritumoral regions of interest ranges based on computed tomography radiomics for the prediction of early recurrence of hepatocellular carcinoma after resection. Quant. Imaging Med. Surg. 13, 6668–6682. 10.21037/qims-23-226 37869280 PMC10585524

[B41] Krassowski M. Das V. Sahu S. K. Misra B. B. (2020). State of the field in multi-omics research: from computational needs to data mining and sharing. Front. Genetics 11, 610798. 10.3389/fgene.2020.610798 33362867 PMC7758509

[B42] Lee D. Son H. Lim L. A. Park K. (2014). Population pharmacokinetic analysis of diurnal and seasonal variations of plasma concentrations of cilostazol in healthy volunteers. Ther. Drug Monit. 36, 771–780. 10.1097/FTD.0000000000000077 24739664

[B43] Li Q. Wang B. Qiu H. Y. Yan X. J. Cheng L. Wang Q. Q. (2021a). Chronic jet lag exacerbates jejunal and colonic microenvironment in mice. Front. Cellular Infection Microbiology 11, 648175. 10.3389/fcimb.2021.648175 34141627 PMC8204051

[B44] Li X. Fan Y. Wang J. Zhou R. Tian L. Wang Y. (2021b). Insulin resistance and metabolic syndrome increase the risk of relapse for fertility preserving treatment in atypical endometrial hyperplasia and early endometrial cancer patients. Front. Oncol. 11, 744689. 10.3389/fonc.2021.744689 34917501 PMC8670892

[B45] Li B. Bao F. Hou Y. Li F. Li H. Deng Y. (2024a). Tissue characterization at an enhanced resolution across spatial omics platforms with deep generative model. Nat. Communications 15, 6541. 10.1038/s41467-024-50837-5 39095360 PMC11297205

[B46] Li H. Cai Z. Wang J. Tang J. Ding W. Lin C. T. (2024b). FedTP: federated learning by transformer personalization. IEEE Transactions Neural Networks Learning Systems 35, 13426–13440. 10.1109/TNNLS.2023.3269062 37220054

[B47] Liang H. Luo H. Sang Z. Jia M. Jiang X. Wang Z. (2024). GREMI: an explainable multi-omics integration framework for enhanced disease prediction and module identification. IEEE Journal Biomedical Health Informatics 28, 6983–6996. 10.1109/JBHI.2024.3439713 39110558

[B48] Lincoln G. A. Andersson H. Loudon A. (2003). Clock genes in calendar cells as the basis of annual timekeeping in mammals--a unifying hypothesis. J. Endocrinol. 179, 1–13. 10.1677/joe.0.1790001 14529560

[B49] Liu S. Li C. Chu M. Zhang W. Wang W. Wang Y. (2022). Associations of forest negative air ions exposure with cardiac autonomic nervous function and the related metabolic linkages: a repeated-measure panel study. Sci. Total Environ. 850, 158019. 10.1016/j.scitotenv.2022.158019 35973547

[B50] Liu N. Deng Q. Hu P. Chang J. Li Y. Zhang Y. (2023). Associations between urban exposome and recurrence risk among survivors of acute myocardial infarction in beijing, China. Environ. Res. 238, 117267. 10.1016/j.envres.2023.117267 37776939 PMC7615203

[B51] Liu B. Li S. Cheng Y. Song P. Xu M. Li Z. (2024a). Distinctive multicellular immunosuppressive hubs confer different intervention strategies for left- and right-sided Colon cancers. Cell Rep. Med. 5, 101589. 10.1016/j.xcrm.2024.101589 38806057 PMC11228667

[B52] Liu Y. Liu W. Lai A. Mei Y. Wang Y. Wei H. (2024b). Multiomic analysis identifies a high-risk subgroup that predicts poor prognosis in t(8;21) acute myeloid leukemia. Blood Cancer Journal 14, 162. 10.1038/s41408-024-01144-1 39284810 PMC11405412

[B53] Liu G. Wang Q. Tian L. Wang M. Duo D. Duan Y. (2024c). Blood-brain barrier permeability is affected by changes in tight junction protein expression at high-altitude hypoxic conditions-this may have implications for brain drug transport. Aaps J. 26, 90. 10.1208/s12248-024-00957-z 39107477

[B54] Luan L. Daoyu Z. (2025). Development of deep learning models for high-resolution exposome mapping and health impact assessment. Front. Public Health 13, 1565471. 10.3389/fpubh.2025.1565471 40535428 PMC12174458

[B55] Manceau H. Amrani K. Peoc'h K. (2017). Personalized medicine, pharmacogenomic and companion biomarker. Ann. Biol. Clin. 75, 631–636. 10.1684/abc.2017.1306 29192598

[B56] Medina J. E. Annapragada A. V. Lof P. Short S. Bartolomucci A. L. Mathios D. (2024). Early detection of ovarian cancer using cell-free DNA fragmentomes and protein biomarkers. Cancer Discov. 15, 105–118. 10.1158/2159-8290.CD-24-0393 39345137 PMC11726017

[B57] Merlo J. Mulinari S. Wemrell M. Subramanian S. V. Hedblad B. (2017). The tyranny of the averages and the indiscriminate use of risk factors in public health: the case of coronary heart disease. SSM Popul. Health 3, 684–698. 10.1016/j.ssmph.2017.08.005 29349257 PMC5769103

[B58] Milliken S. V. Wassall H. Lewis B. J. Logie J. Barker R. N. Macdonald H. (2012). Effects of ultraviolet light on human serum 25-hydroxyvitamin D and systemic immune function. J. Allergy Clin. Immunol. 129, 1554–1561. 10.1016/j.jaci.2012.03.001 22502796

[B59] Mirza B. Wang W. Wang J. Choi H. Chung N. C. Ping P. (2019). Machine learning and integrative analysis of biomedical big data. Genes 10, 87. 10.3390/genes10020087 30696086 PMC6410075

[B60] Moyer A. M. Matey E. T. Miller V. M. (2019). Individualized medicine: sex, hormones, genetics, and adverse drug reactions. Pharmacol. Research and Perspectives 7, e00541. 10.1002/prp2.541 31844524 PMC6897337

[B61] Mukherji A. Jühling F. Simanjuntak Y. Crouchet E. Del Zompo F. Teraoka Y. (2024). An atlas of the human liver diurnal transcriptome and its perturbation by hepatitis C virus infection. Nat. Commun. 15, 7486. 10.1038/s41467-024-51698-8 39209804 PMC11362569

[B62] Nurmohamed N. S. Belo Pereira J. P. Hoogeveen R. M. Kroon J. Kraaijenhof J. M. Waissi F. (2022). Targeted proteomics improves cardiovascular risk prediction in secondary prevention. Eur. Heart J. 43, 1569–1577. 10.1093/eurheartj/ehac055 35139537 PMC9020984

[B63] Olivier M. Asmis R. Hawkins G. A. Howard T. D. Cox L. A. (2019). The need for multi-omics biomarker signatures in precision medicine. Int. J. Mol. Sci. 20, 4781. 10.3390/ijms20194781 31561483 PMC6801754

[B64] Park T. Gu P. Kim C. H. Kim K. T. Chung K. J. Kim T. B. (2023). Artificial intelligence in urologic oncology: the actual clinical practice results of IBM watson for oncology in South Korea. Prostate International 11, 218–221. 10.1016/j.prnil.2023.09.001 38196551 PMC10772151

[B65] Pastorino R. Loreti C. Giovannini S. Ricciardi W. Padua L. Boccia S. (2021). Challenges of prevention for a sustainable personalized medicine. J. Pers. Med. 11, 311. 10.3390/jpm11040311 33923579 PMC8073054

[B66] Plasqui G. Kester A. D. Westerterp K. R. (2003). Seasonal variation in sleeping metabolic rate, thyroid activity, and leptin. Am. J. Physiol. Endocrinol. Metab. 285, E338–E343. 10.1152/ajpendo.00488.2002 12857676

[B67] Potter G. D. M. Wood T. R. (2020). The future of shift work: circadian biology meets personalised medicine and behavioural science. Front. Nutrition 7, 116. 10.3389/fnut.2020.00116 32850937 PMC7426458

[B68] Price N. D. Magis A. T. Earls J. C. Glusman G. Levy R. Lausted C. (2017). A wellness study of 108 individuals using personal, dense, dynamic data clouds. Nat. Biotechnology 35, 747–756. 10.1038/nbt.3870 28714965 PMC5568837

[B69] Ratiner K. Ciocan D. Abdeen S. K. Elinav E. (2024). Utilization of the microbiome in personalized medicine. Nat. Rev. Microbiol. 22, 291–308. 10.1038/s41579-023-00998-9 38110694

[B70] Riggs D. W. Yeager R. A. Bhatnagar A. (2018). Defining the human envirome: an omics approach for assessing the environmental risk of cardiovascular disease. Circ. Res. 122, 1259–1275. 10.1161/CIRCRESAHA.117.311230 29700071 PMC6398443

[B71] Rudin C. (2019). Stop explaining black box machine learning models for high stakes decisions and use interpretable models instead. Nat. Machine Intelligence 1, 206–215. 10.1038/s42256-019-0048-x 35603010 PMC9122117

[B72] Saliba A. Du Y. Feng T. Garmire L. (2024). Multi-omics integration in nephrology: advances, challenges, and future directions. Seminars Nephrology 44, 151584. 10.1016/j.semnephrol.2025.151584 40216576 PMC12278776

[B73] Sato S. Dyar K. A. Treebak J. T. Jepsen S. L. Ehrlich A. M. Ashcroft S. P. (2022). Atlas of exercise metabolism reveals time-dependent signatures of metabolic homeostasis. Cell Metab. 34, 329–345.e8. 10.1016/j.cmet.2021.12.016 35030324 PMC13189211

[B74] Savikj M. Stocks B. Sato S. Caidahl K. Krook A. Deshmukh A. S. (2022). Exercise timing influences multi-tissue metabolome and skeletal muscle proteome profiles in type 2 diabetic patients - a randomized crossover trial. Metabolism Clinical Experimental 135, 155268. 10.1016/j.metabol.2022.155268 35908579

[B75] Schadt E. E. (2009). Molecular networks as sensors and drivers of common human diseases. Nature 461, 218–223. 10.1038/nature08454 19741703

[B76] Shao J. Ma J. Zhang Q. Li W. Wang C. (2023). Predicting gene mutation status *via* artificial intelligence technologies based on multimodal integration (MMI) to advance precision oncology. Semin. Cancer Biol. 91, 1–15. 10.1016/j.semcancer.2023.02.006 36801447

[B77] Sharma D. Adnan D. Abdel-Reheem M. K. Anafi R. C. Leary D. D. Bishehsari F. (2024). Circadian transcriptome of pancreatic adenocarcinoma unravels chronotherapeutic targets. JCI Insight 9, e177697. 10.1172/jci.insight.177697 38716727 PMC11141942

[B78] Siddiqui N. A. Ventrola A. J. Hartman A. R. Konare T. Kamble N. S. Thomas S. C. (2023). An engineered probiotic platform for cancer epitope-independent targeted radionuclide therapy of solid tumors. Adv. Healthcare Materials 12, e2202870. 10.1002/adhm.202202870 36913614 PMC10497710

[B79] Sinturel F. Chera S. Brulhart-Meynet M. C. Montoya J. P. Stenvers D. J. Bisschop P. H. (2023). Circadian organization of lipid landscape is perturbed in type 2 diabetic patients. Cell Rep. Med. 4, 101299. 10.1016/j.xcrm.2023.101299 38016481 PMC10772323

[B80] Sun J. Liu Y. Zhao J. Lu B. Zhou S. Lu W. (2024). Plasma proteomic and polygenic profiling improve risk stratification and personalized screening for colorectal cancer. Nat. Commun. 15, 8873. 10.1038/s41467-024-52894-2 39402035 PMC11473805

[B81] Sung J. (2023). Artificial intelligence in medicine: ethical, social and legal perspectives. Ann. Acad. Med. Singap. 52, 695–699. 10.47102/annals-acadmedsg.2023103 38920162

[B82] Thomas S. C. Madaan T. Kamble N. S. Siddiqui N. A. Pauletti G. M. Kotagiri N. (2022). Engineered bacteria enhance immunotherapy and targeted therapy through stromal remodeling of tumors. Adv. Healthcare Materials 11, e2101487. 10.1002/adhm.202101487 34738725 PMC8770579

[B83] Ting D. S. W. Pasquale L. R. Peng L. Campbell J. P. Lee A. Y. Raman R. (2019). Artificial intelligence and deep learning in ophthalmology. Br. Journal Ophthalmology 103, 167–175. 10.1136/bjophthalmol-2018-313173 30361278 PMC6362807

[B84] Tsuruta A. Shiiba Y. Matsunaga N. Fujimoto M. Yoshida Y. Koyanagi S. (2022). Diurnal expression of PD-1 on tumor-associated macrophages underlies the dosing time-dependent antitumor effects of the PD-1/PD-L1 inhibitor BMS-1 in B16/BL6 melanoma-bearing mice. Mol. Cancer Res. 20, 972–982. 10.1158/1541-7786.MCR-21-0786 35190830 PMC9381128

[B85] Wang Q. (2025). From digits towards digitization: the past, present, and future of traditional Chinese medicine. Digit. Chin. Med. 8, 4–19. 10.1016/j.dcmed.2025.03.002

[B86] Wang X. Ning Y. Li C. Gong Y. Huang R. Hu M. (2021). Alterations in the gut microbiota and metabolite profiles of patients with kashin-beck disease, an endemic osteoarthritis in China. Cell Death and Disease 12, 1015. 10.1038/s41419-021-04322-2 34711812 PMC8553765

[B87] Wang R. Peng X. Yuan Y. Shi B. Liu Y. Ni H. (2024). Dynamic immune recovery process after liver transplantation revealed by single-cell multi-omics analysis. Innov. (Camb) 5, 100599. 10.1016/j.xinn.2024.100599 38510071 PMC10952083

[B88] Watanabe K. Wilmanski T. Diener C. Earls J. C. Zimmer A. Lincoln B. (2023). Multiomic signatures of body mass index identify heterogeneous health phenotypes and responses to a lifestyle intervention. Nat. Medicine 29, 996–1008. 10.1038/s41591-023-02248-0 36941332 PMC10115644

[B89] Wei J. Wang A. Yu P. Sun Y. Wu W. Zhang Y. (2025). Integrating multi-omics and machine learning strategies to explore the “gene-protein-metabolite” network in ischemic heart failure with Qi deficiency and blood stasis syndrome. Chin. Medicine 20, 93. 10.1186/s13020-025-01151-9 40671145 PMC12269143

[B90] Weng H. Deng L. Wang T. Xu H. Wu J. Zhou Q. (2024). Humid heat environment causes anxiety-like disorder *via* impairing gut microbiota and bile acid metabolism in mice. Nat. Commun. 15, 5697. 10.1038/s41467-024-49972-w 38972900 PMC11228019

[B91] Wild C. P. (2012). The exposome: from concept to utility. Int. Journal Epidemiology 41, 24–32. 10.1093/ije/dyr236 22296988

[B92] Xin H. Deng F. Zhou M. Huang R. Ma X. Tian H. (2021). A multi-tissue multi-omics analysis reveals distinct kineztics in entrainment of diurnal transcriptomes by inverted feeding. iScience 24, 102335. 10.1016/j.isci.2021.102335 33889826 PMC8050734

[B93] Xu L. Wu X. Long L. Li S. Huang M. Li M. (2025). TGR5 attenuates DOCA-salt hypertension through regulating histone H3K4 methylation of ENaC in the kidney. Metabolism Clinical Experimental 165, 156133. 10.1016/j.metabol.2025.156133 39824478

[B94] Yan S. Y. Ma L. H. Yang W. X. (2024). Altitude and prognosis after PCI: a propensity score-matched analysis. Heliyon 10, e33577. 10.1016/j.heliyon.2024.e33577 39091961 PMC11292508

[B95] Yang Y. Lin M. Zhao H. Peng Y. Huang F. Lu Z. (2024). A survey of recent methods for addressing AI fairness and bias in biomedicine. J. Biomedical Informatics 154, 104646. 10.1016/j.jbi.2024.104646 38677633 PMC11129918

[B96] You J. Wang L. Wang Y. Kang J. Yu J. Cheng W. (2024a). Prediction of future parkinson disease using plasma proteins combined with clinical-demographic measures. Neurology 103, e209531. 10.1212/WNL.0000000000209531 38976826

[B97] You L. Kou J. Wang M. Ji G. Li X. Su C. (2024b). An exposome atlas of serum reveals the risk of chronic diseases in the Chinese population. Nat. Communications 15, 2268. 10.1038/s41467-024-46595-z 38480749 PMC10937660

[B98] Zeybel M. Arif M. Li X. Altay O. Yang H. Shi M. (2022). Multiomics analysis reveals the impact of microbiota on host metabolism in hepatic steatosis. Adv. Sci. (Weinh) 9, e2104373. 10.1002/advs.202104373 35128832 PMC9008426

[B99] Zhang Q. Du X. Li H. Jiang Y. Zhu X. Zhang Y. (2022). Cardiovascular effects of traffic-related air pollution: a multi-omics analysis from a randomized, crossover trial. J. Hazardous Materials 435, 129031. 10.1016/j.jhazmat.2022.129031 35523096

[B100] Zhao Y. Tang J. Jiang K. Liu S. Y. Aicher A. Heeschen C. (2023a). Liquid biopsy in pancreatic cancer - current perspective and future outlook. Biochim. Biophys. Acta Rev. Cancer 1878, 188868. 10.1016/j.bbcan.2023.188868 36842769

[B101] Zhao Q. Chen Y. Huang W. Zhou H. Zhang W. (2023b). Drug-microbiota interactions: an emerging priority for precision medicine. Signal Transduction Targeted Therapy 8, 386. 10.1038/s41392-023-01619-w 37806986 PMC10560686

[B102] Zhao F. Zhang C. Geng B. (2024). Deep multimodal data fusion, 56. Article 216.

[B103] Zhou S. Guan C. Deng S. Zhu Y. Yang W. Zhang X. (2025). A novel sequence-based transformer model architecture for integrating multi-omics data in preterm birth risk prediction. NPJ Digital Medicine 8, 536. 10.1038/s41746-025-01942-2 40835718 PMC12368083

